# Targeting HSP90 in ovarian cancers with multiple receptor tyrosine kinase coactivation

**DOI:** 10.1186/1476-4598-10-125

**Published:** 2011-09-30

**Authors:** Yisheng Jiao, Wenbin Ou, Fanguo Meng, Haimeng Zhou, Aiyuan Wang

**Affiliations:** 1 Department of Obstetrics and Gynecology, Shengjing Hospital of China Medical University, Shenyang, P. R. China; 2 Yangtze Delta Region Institute of Tsinghua University, Jiaxing, Zhejiang, P. R. China; 3 Department of Ophthalmology, Shengjing Hospital of China Medical University, Shenyang, P. R. China

**Keywords:** Ovarian Cancer, Tyrosine Kinases, coactivation, HSP90

## Abstract

**Background:**

Ovarian cancer has the highest mortality rate of all gynecologic malignancy. The receptor tyrosine kinases (RTKs), including EGFR, ERBB2, PDGFR, VEGFR and MET, are activated in subsets of ovarian cancer, suggesting that these kinases might represent novel therapeutic targets. However, clinical trials have not or just partially shown benefit to ovarian cancers treated with EGFR, ERBB2, or PDGFR inhibitors. Despite multiple RTK activation in ovarian cancer pathogenesis, it is unclear whether transforming activity is dependent on an individual kinase oncoprotein or the coordinated activity of multiple kinases. We hypothesized that a coordinated network of multi-RTK activation is important for the tumorigenesis of ovarian cancers.

**Results:**

Herein, we demonstrate co-activation of multiple RTKs (EGFR, ERBB2, ERBB4, MET and/or AXL) in individual ovarian cancer cell lines and primary tumors. We also show that coordinate inhibition of this multi-kinase signaling has substantially greater effect on ovarian cancer proliferation and survival, compared to inhibition of individual activated kinases. The inhibition of this multi-RTK signaling by HSP90 suppression results in profound pro-apoptotic and anti-proliferative effects, and is associated with the inactivation of RTK downstream PI3-K/AKT/mTOR and RAF/MAPK signaling.

**Conclusion:**

These studies suggest that anti-multiple RTK strategy could be useful in the treatment of ovarian cancer.

## Background

Ovarian cancer is a leading cause of cancer death among women in Western Europe and the United States, which has the highest mortality rate of all gynecologic malignancy [1,2]. Ovarian cancer histologic subtypes include epithelioid (serous, endometrioid, mucinous, clear cell and undifferentiated) and non-epitheliod [3], of which the epithelioid subtype accounts for 90% of ovarian malignancies [4]. Although more than 70% patients have increased 5-year survival rates after surgery followed by chemotherapy and second-line therapies [5], the low overall cure rates and the intolerable side effects of systemic chemotherapy asks for the development of novel and more effective pharmacological interventions. An improved understanding of ovarian cancer biology - including crucial growth factor signaling pathways - is needed for the identification of biologically rational targets for novel therapies.

The increasing evidences suggest that receptor tyrosine kinase (RTK) activation participates in the oncogenic progression from nonneoplastic mesothelial lining of the ovaries or the fallopian tube epithelium to epithelial ovarian cancer. Epidermal growth factor receptor (EGFR) is amplified in approximately 4%-22% of ovarian cancer and activating EGFR mutations is rare with a frequency of 4% or less [6-8]. EGFR upregulation is detected in ~60% ovarian cancer and associated with increased tumor cell proliferation, advanced tumor grades and poor patient prognosis [6,7]. Furthermore, the EGFR small molecular inhibitors gefitinib and erlotinib inhibited EGFR-mediated AKT and MAPK phosphorylation and decreased tumor cell proliferation in some ovarian cancer cell lines and tumor xenograft models [3]. ERBB2 overexpression and amplification are present in a subset of epithelial ovarian cancer and serous carcinoma [9,10]. Anti-ERBB2 Trastuzumab and lapatinib inhibited the proliferation and tumor growth in ovarian cancers with ERBB2 upregulation [3,9,11]. More recently, an activated ERBB3/NRG1 autocrine loop has been demonstrated to support tumor cell proliferation in a subset of primary ovarian cancers and ovarian cancer cell lines [12]. The MET receptor tyrosine kinase and its ligand (hepatocyte growth factor, HGF) are highly expressed in ovarian cancers, and MET inactivation by small molecular inhibitor and siRNA reduced tumor burden and metastasis in nude mice with ovarian cancer [13,14]. EPHA2 is overexpressed in many types of human cancer but is absent in normal epithelial tissues [15]. EPHA2 inhibition by dasatinib or a novel immunoconjugate containing an anti-EPHA2 monoclonal antibody linked to a chemotherapeutic agent, shows antitumor activity against EPHA2-positive ovarian cancer cell lines and mouse tumor models [15,16]. Platelet derived growth factor receptor (PDGFR) is expressed in 50-80% of ovarian cancers [17]. High expression of PDGFR has been correlated with aggressive tumor phenotypes including high proliferation index and advanced histologic grade [18]. PDGFR inactivation by both RNAi and a neutralizing antibody, results in significant anti-proliferative effects in ovarian cancer cells [19]. High expression of VEGF (vascular endothelial growth factor) and its receptors (VEGFR-1, -2, and-3) has been associated with poor prognosis in ovarian cancer [20,21]. Anti-angiogenic Pazopanib or sunitinib suppressed tumor growth in preclinical ovarian cancer models [2]. The AXL receptor tyrosine kinase protein, and its ligand Gas 6 (growth arrest-specific gene 6) are expressed significantly higher in ovarian cancers than in normal ovaries, although its role in the tumorigenesis of ovarian cancer needs further studies [22].

In addition, numerous evidences have indicated the association between *TP53 *mutations in ovarian cancer and prognosis. Most high-grade serous carcinomas are characterized by *TP53 *mutations and lack of mutations of *KRAS*, *BRAF*, or *ERBB2 *[23]. Mutant p53 is almost invariably present and plays a crucial role in the molecular pathogenesis of high grade serous carcinoma [24].

In recent years, RTK-targeted cancer therapies - for example, anti-ERBB2 in breast cancer [25], anti-KIT and PDGFA in gastrointestinal stromal tumors (GISTs) [26], anti-BCR-ABL in chronic myelogenous leukemia [27] and anti-EGFR in non-small-cell lung cancer [28] - have seen widespread clinical use. However, despite the abovementioned evidence for tyrosine kinase activation in ovarian cancer pathogenesis, targeted anti-kinase therapies just had only minimal or partial clinical response in patients with ovarian cancer [2,3]. In the current studies we demonstrate the simultaneous activation of multiple RTKs - including EGFR, ERBB2, MET, and/or AXL - in individual ovarian cancer cell lines and primary tumors. We also showed that HSP90 inhibition is a compelling approach to inactivate multiple RTKs. The inhibition of multiple RTKs had superior effect in maximizing apoptosis and anti-proliferation compared to the inactivation of any single RTK inhibition in these models. These studies highlight multiple RTK inactivation by HSP90 inhibition as a novel therapeutic strategy in ovarian cancer.

## Materials and methods

### Antibodies and reagents

Monoclonal antibodies to EGFR (immunoprecipitation), phosphotyrosine (PY99), p53 and PCNA and polyclonal antibodies to EGFR, ERBB4, MET and AXL were from Santa Cruz Biotechnology (Santa Cruz, CA, USA). Polyclonal antibodies to AKT and cleaved caspase 8 were from Cell Signaling Technology (Beverly, MA, USA). Antibodies to ERBB2, MAPK, and PARP were from Zymed/Invitrogen Laboratories (Invitrogen life Technologies, Carlsbad, CA, USA). Phospho-specific antibodies and monoclonal antibody to S6 were from Cell Signaling Technology. Monoclonal antibody to p27 was from BD Transduction Laboratories (San Jose, CA). Monoclonal antibody to β-actin, lentiviral AXL shRNA constructs, and polybrene were from Sigma-Aldrich (St, Louis, MO, USA). Targeting AXL sequences: GCTGTGAAGACGATGAAGATT (shRNA1); CTTTAGGTTCTTTGCTGCATT (shRNA2); CGAAAGAAGGAGACCCGTTAT (shRNA3). Control scrambled shRNA: AAGUUCAGGUCGAUAUGUGCA.

Human Phospho-RTK Array Kit was from R&D Systems (Minneapolis, MN, USA). 17-allyloamino-17-demethoxygeldanamycin (17-AAG) and gefitinib were obtained from LC Laboratories (Woburn, MA, USA) and PHA-665752 was from Tocris Biosciences (St. Louis, MO, USA). AUY922 was obtained from Selleck (Shanghai, China). All inhibitors were reconstituted in DMSO. Protein A- and Protein G-sepharose beads were purchased from Zymed Laboratories (Invitrogen Life Technologies).

### Ovarian cancer cell Lines

Ovarian cancer cell lines derived from serous (SKOV3 and OVCA429), and clear cell (ES2) adenocarcinomas were used in this study. Ovarian cancer cells are kind gifts from Dr. Ross Berkowitz in the Laboratory of Gynecologic Oncology at Brigham and Women's Hospital and Harvard Medical School. Ovarian cancer cell lines were maintained in RPMI 1640 with 10% fetal bovine serum (FBS) containing penicillin/streptomycin and L-glutamine.

### Frozen tumor specimens

All frozen tumor specimens (Chinese patients) were obtained from Shengjing Hospital of China Medical University. These studies were approved by the China Medical University Institutional Review Board, under a discarded tissues protocol. p1, p2, p3, p8, p9, p10, p11, p12, p13, and p14 were epithelioid type ovarian cancers; p4, p6, and p15 were non-epithelioid type ovarian cancers; and p5 and p7 were borderline mucinous cystadenomas.

### Phospho-RTK array analysis

The Human Phospho-RTK Array Kit was used to determine the relative levels of tyrosine phosphorylation of 42 distinct RTKs. Phospho-RTK arrays were performed as product protocol. Briefly, After serum starved for 2 h, SKOV3, OVCA429, and ES2 lysates were prepared using lysis buffer (1% NP-40, 50 mM Tris-HCl pH 8.0, 100 mM sodium fluoride, 30 mM sodium pyrophosphate, 2 mM sodium molybdate, 5 mM EDTA, 2 mM sodium orthovanadate) containing protease inhibitors (10 μg/mL aprotinin, 10 μg/mL leupeptin, 1 mM phenylmethylsulfonyl fluoride). The arrays were incubated with 500 μg of protein lysates overnight at 4°C after blocking 1 h by using Array Buffer1. The arrays were washed and incubated with a horseradish peroxidase-conjugated phospho-tyrosine detection antibody (1:5000). Detection was by chemiluminescence (ImmobilonTM Western, Millipore Corporation, MA), captured using a FUJI LAS 1000-plus chemiluminescence imaging system.

### Protein lysate preparations and immunoblotting

Phosphorylation of RTK and downstream signaling was performed by immunoblotting ovarian cancer cell lysates after treatment with 17-AAG or AUY922 for 4 h in serum free medium. Total RTK expression, proliferation and apoptosis marker immunoblotting studies were performed using cell lysates after 48 h treatment in serum containing media. Frozen tumor samples were diced into small pieces in cold lysis buffer on dry ice and homogenized using a Tissue Tearor (Biospec Products, Inc. USA) for three or five seconds, 3-5 times, on ice, and the cell lysate was then rocked for overnight at 4°C. Lysates were spined down by centrifugation at 14,000 rpm for 30 min at 4°C, and lysate protein concentrations were determined using a Bio-Rad protein assay (Bio-Rad Laboratories Hercules, CA, USA). Electrophoresis and immunoblotting were performed as described previously [29], with hybridization signals detected by chemiluminescence (ImmobilonTM Western, Millipore Corporation, MA) and captured using a FUJI LAS1000-plus chemiluminescence imaging system.

### Immunoprecipitation

Ovarian cancer cell lysates were prepared after serum starved for 2 h or treatment with 1 μM 17-AAG in serum free medium for 6 h. One mg of protein lysate was precleared for 30 min using 30 μl of protein G or protein A beads at 4°C. Two μg of EGFR, ERBB2, ERBB4, MET, or AXL antibody was added to the supernatants and rocked for 2-4 h at 4°C. Then 25 μL of sepharose-protein G or -protein A beads were added and rocked overnight at 4°C, then centrifuged at 14,000 rpm for 2 min at 4°C, after which the sepharose beads were washed 3 times with 750 μL of IP buffer (25 min/each time) and once with 750 μL 10 mM Tris-Cl buffer (pH7.6). Loading buffer (20 μL) was added to the beads and boiled for 5 min at 95°C.

### Lentivirus preparation

Lentivirus preparations were produced by cotransfecting empty vector pLKO.1puro with AXL shRNA, and helper virus packaging plasmids pCMV Δ R8.91 and pMD.G (at a 10:10:1 ratio) into 293T cells. Transfections were carried out using lipofectamine and PLUS reagent. Lentiviruses were harvested at 24, 36, 48, and 60 h post-transfection. Virus was frozen at -80°C in appropriately sized aliquots for infection.

### Cell Culture and Virus infection

OVCA429 cells were cultured in RPMI 1640 medium with 10% fetal bovine serum and seeded in six-well plates. Lentiviral shRNA infections were carried out in the presence of 8 μg/mL polybrene. Cells were lysed for western blot analysis at 72 h post-infection.

### Cell proliferation and apoptosis assays

SKOV3, OVCA429, and ES2 cells were plated at 4, 000 cells/well in a 96-well flat-bottomed plate (Falcon, Lincoln NJ) and cultured in media for 24 hours before being infected with lentiviral AXL shRNAs or different inhibitors, which included gefitinib (1 μM), PHA-665752 (1 μM) alone or combination, 17-AAG (0.1, 0.25, 0.5 and 1 μM), and AUY922 (0.1, 0.25, 0.5 and 1 μM). Cell viability and apoptosis were determined after treatment with inhibitors for 24 hours, and 3 and 6 days using the Caspase-Glo 3/7 assay kit and the CellTiter-Glo luminescent assay from Promega (Madison, WI), and measured using a Veritas™ Microplate Luminometer (Turner Biosystems, Sunnyvale, CA). The data were normalized to the control group (scrambled shRNA or DMSO). All experimental points were set up in four replicate wells and independently performed in triplicate.

Apoptosis was also evaluated using PE Annexin V Apoptosis Detection Kit I (BD Pharmingen™, USA). Briefly, SKOV3, OVCA429, and ES2 cells in 6-well plates were treated with 17-AAG or AUY922 for 48 hours, trypsinized and washed twice with cold Hanks Balanced Salt Solution and treated with 5 μl of PE Annexin V and 5 μl 7-AAD in 1X Binding Buffer for 15 minutes at RT (25°C) in dark. The stained cells were analyzed in a flow cytometer within 1 hour and ModFit LT (Machitosh) was used to analyze the data.

### Cell cycle analysis

SKOV3, OVCA429, and ES2 cells in 6-well plates were treated with 17-AAG or AUY922 for 48 hours, then trypsinized and washed once with Hanks Balanced Salt Solution. For nuclear staining, cells were fixed by 70% ethanol for 24 h. A propidium iodide (PI)-containing solution (Roche) was added to the cells and incubated for 15 minutes at 37°C. The cell suspension was analyzed on a flow cytometer within 48 hours and ModFit LT (Machitosh) was used to fit the data.

### Statistical analysis

Student's *t*-tests was performed to analyze data from cells treated with control DMSO or 17-AAG/AUY922, as well as cells treated with control scrambled shRNA+DMSO or combination of gefitinib, PHA, and AXL shRNA1/AXL shRNA2. Statistically significant differences were defined as *P *< 0.05 and *P *< 0.01.

## Results

### Expression and activation of multiple RTKs in ovarian cancer cells

**Figure 1:**
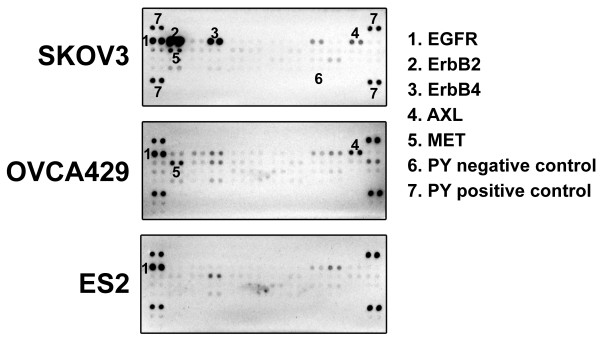
**Coactivation of multiple RTKs in ovarian cancer cell lines**. Total cell lysates (500 μg) from SKOV3, OVCA429, and ES2 cells that were maintained under serum starved medium for 2 h were subjected to phospho-RTK array. Positive duplicated spots for individual RTK were numbered and indicated on the right of the array. Two dots at each corner indicate positive controls and eight dots at the lower right indicate negative controls.

By phospho-RTK assays, the expression and activation of EGFR, ERBB2, ERBB4 and MET were activated in SKOV3 cells, and EGFR, MET and AXL in OVCA429 cells, and EGFR in ES2 cells under serum starved medium condition (Figure 1).

**Figure 2:**
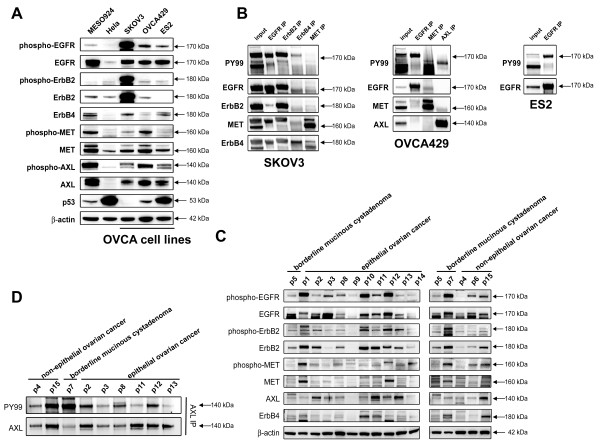
**Immunoblotting and immunoprecipitation evatulations of multi-RTK coactivation in ovarian cancer cell lines and primay tumors**. **A**) Imunoblotting evaluations of EGFR, ERBB2, ERBB4, MET, AXL and p53 expression and/or activation in ovarian cancer cell lines (SKOV3, OVCA429 and ES2), mesothelioma cell line (MESO924, positive control), and Hela cell line (negative control). **B**) The activation of EGFR, ERBB2, ERBB4, MET, and AXL was validated by immunoprecipitation followed by phosphotyrosine and EGFR, ERBB2, ERBB4, MET, and AXL immunoblotting in ovarian cancer cell lines (SKOV3, OVCA429 and ES2). **C**) Imunoblotting evaluations of EGFR, ERBB2, ERBB4, MET, and AXL expression and/or activation in ovarian cancer primary frozen tumors (p1-p15). **D**) The activation of AXL was validated by immunoprecipitation followed by phosphotyrosine and AXL immunoblotting in ovarian cancer primary frozen tumors.

Activation and/or expression of multiple RTK EGFR, ERBB2, ERBB4, MET, and AXL in ovarian cancer cell lines were further validated by immunoblotting with phospho-specific antibodies; As shown in Figure 2A, EGFR, ERBB2, ERBB4, and MET in SKOV3, EGFR, MET, and AXL in OVCA429, and EGFR in ES2 were strongly phosphorylated (Figure 2A). EGFR, MET, and AXL activation in the ovarian cancer lines was comparable to that in MESO924 cells (Figure 2A), which are known to feature strong activation of these RTK [30]. By contrast, activation of EGFR, ERBB2, MET, and AXL was weak-to-undetectable in Hela cells. Co-activation and co-expression of multiple RTKs were further confirmed in these cells by immunoprecipitation with RTK-specific antibodies and immunoblotted with phosphotyrosine (PY99) antibody (Figure 2B). Immunoblotting showed strong and moderate p53 expression in ES2 and OVCA429, respectively, whereas p53 was undetectable in SKOV3 (Figure 2A).

We further evaluated the simultaneous expression/activation of multiple RTKs by immunoblotting and immunoprecipitation in 15 primary ovarian tumors including 3 non-epithelial ovarian tumor (p4, p6, and p15), and 12 epithelial ovarian tumors (p1-p3, and p8-p14) (Figure 2C and 2D). Receptor EGFR, ERBB2, MET, and AXL were strongly co-activated in most primary ovarian tumors (Figure 2C and 2D).

**Figure 3:**
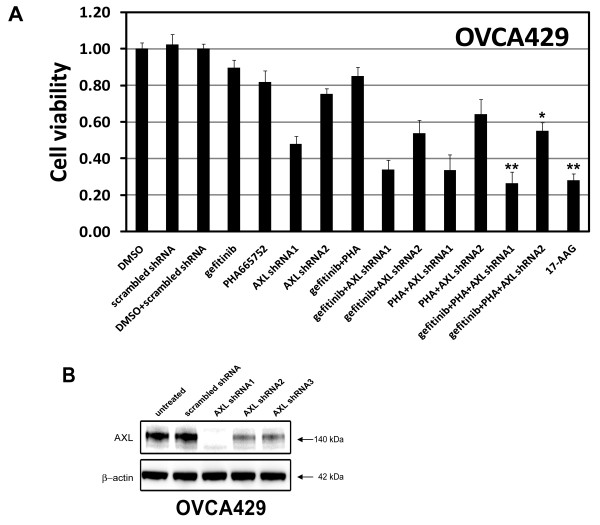
**Consequences of single vs. combination tyrosine kinase inhibitor treatments on ovarian cancer viability**. **A**) Cell viability, in ovarian cancer cell line OVCA429, was evaluated by the CellTiter-Glo luminescence assay after 72 h treatment with EGFR (1 μM gefitinib), MET (1 μM PHA665752), or AXL (AXL shRNA1 and AXL shRNA2) inhibitor alone and together, and HSP90 inhibitors (1 μM 17-AAG). The data were normalized to the DMSO control, and represent the mean values (± s.d.) of quadruplicate cultures (*p < 0.05, **P < 0.01, n = 3). **B**) Imunoblotting evaluations of AXL expression in OVCA429 cells post-infection AXL shRNA at 72 h.

We next compared the inhibitionary effect of tumor cell proliferation between HSP90 inhibitor 17-AAG and various individual kinase inhibitors. EGFR, MET, and AXL signaling pathways in OVCA429 cells were blocked individually by EGFR inhibitor gefitinib, MET inhibitor PHA665752, or shRNA specific to AXL; various combination of kinase inhibitors were also performed, As shown in Figure 3A, the most striking reduction in cell viability was seen in cells treated with 17-AAG or combination of all 3 kinase inhibitors with ~75% cell decrease observed. EGFR and MET inhibitors alone or together had mild or little effects on cell viability. AXL inhibition by lentiviral shRNA1 and shRNA2 resulted in 50% and 25% inhibition of cell viability in OVCA429, respectively, whereas combination of EGFR/MET and AXL inhibition resulted in 65% reduction in viability (Figure 3A). The AXL shRNA-mediated knockdown resulted in ~95% (AXL shRNA1) and 60% (AXL shRNA2 and shRNA3) decrease of AXL protein expressionin OVCA429 (Figure 3B).

### Inactivation of multi-RTKs and downstream intermediates by HSP90 inhibition

The observation that individual RTK inhibitors have little effect on cell viability (Figure 3A), suggested that activation of any one RTK is insufficient to sustain ovarian cancer growth and/or survival. Because the effect of HSP90 inhibition on cell viability were comparable, or greater than combination of EGFR, MET, and AXL suppression (Figure 3A), and multiple RTK EGFR, ERBB2, MET, and/or AXL were simutaneously activated in individual ovarian cancer cells (Figure 1 and 2), we hypothesized that the HSP90 inhibition collectively inactivated RTK downstream intermediates including PI3-K/AKT/mTOR and RAF/MAPK signaling. HSP90 has crucial roles in maintaining the conformation and stability of many activated RTKs, including EGFR, ERBB2, and MET [31]. We therefore evaluated whether HSP90 inhibition collectively inactived multiple RTKs and their downstream signaling pathways, which have been implicated in maintaining proliferation and survival in ovarian cancers [3,13].

**Figure 4:**
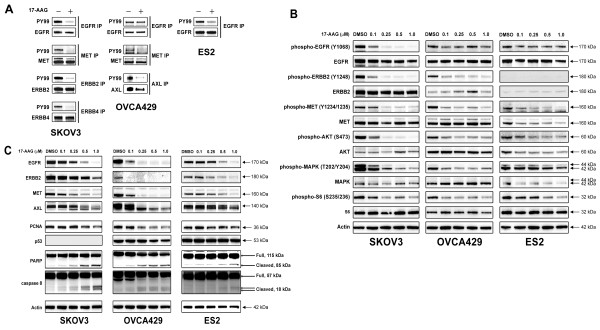
**Inactivation of multi-RTKs and downstream intermediates by HSP90 inhibition**. **A**) Inhibition of EGFR, ERBB2, ERBB4, AXL and MET phosphorylation in cell immunoprecipitates after 4 h 17-AAG (1 μM) treatment in serum-free medium. **B**) Immunoblotting evaluated effects of HSP90 inhibitor 17-AAG on RTK EGFR, ERBB2, and MET, and intermediate AKT, S6 and MAPK activation, after 4 h treatment in serum-free medium. **C**) Effects of HSP90 inhibitor 17-AAG on EGFR, ERBB2, MET, AXL, PCNA, p53, Caspase 8 and PARP expression in ovarian cancer lines, after 48 h 17-AAG treatment in serum-containing medium. β-actin stain is a loading control.

In SKOV3 and OVCA429, phosphoRTK evaluations of ovarian cancer total cell lysates demonstrated co-activation of multiply RTKs (Figure 1A). EGFR, ERBB2, ERBB4, and MET immunoprecipitations in SKOV3, EGFR, MET, and AXL immunoprecipitations in OVCA429, and EGFR immunoprecipitation in ES2, from DMSO vs. 17-AAG treated ovarian cancer cells confirmed 17-AAG mediated inhibition of multi-RTK tyrosine phosphorylation, as demonstrated by phosphotyrosine (PY99) immunostaining (Figure 4A). Immunoblotting evaluations of ovarian cancer total cell lysates also demonstrated inactivation of EGFR, ERBB2, and MET after 17-AAG treatment (Figure 4B). Inhibition of total EGFR, ERBB2, MET and AXL expression was seen in all ovarian cancer cell lines after treatment with 17-AAG in serum-containing medium for 48 hours (Figure 4C). AKT and S6 were substantially and dose-dependently inactivated in all three ovarian cancer cell lines after HSP90 inhibition, whereas MAPK was inactivated in two of the ovarian cancer lines (SKOV3 and OVCA429) (Figure 4B).

### HSP90 regulation of ovarian cancer proliferation

**Figure 5:**
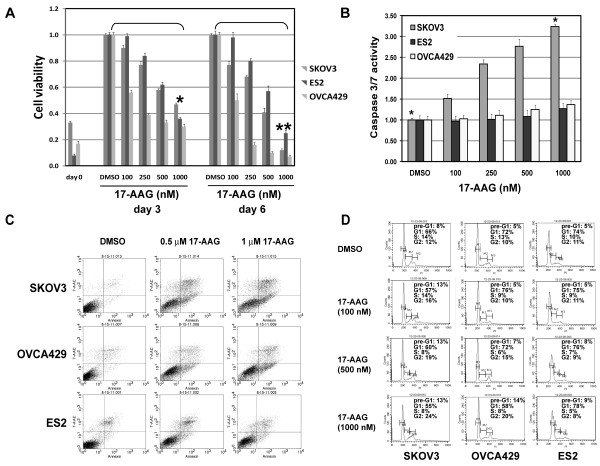
**HSP90 regulation of ovarian cancer proliferation and survival**. **A**) Ovarian cancer cell viability was determined by the CellTiter-Glo assay after 3 and 6 day treatment with HSP90 inhibitor, 17-AAG. The data were normalized to the DMSO control, and represent the mean values (± s.d.) of quadruplicate cultures (*p < 0.05, **P < 0.01, n = 3). **B**) Apoptosis was evaluated in ovarian cancer cell lines, at 24 h after treatment with 17-AAG. Caspase 3/7 activity was measured using a Caspase-Glo luminescence assay. The data were normalized to the DMSO control, and represent the mean values (± s.d.) from quadruplicate cultures (*p < 0.01, n = 3). **C**) Apoptosis analyses following 17-AAG (0.5 and 1 μM) treatment for 48 hours by using PE Annexin V Apoptosis Detection Kit I. **D**) Cell cycle analyses after 48 hours inhibitor treatment in serum-containing medium.

We extended our studies of HSP90 inhibition on proliferation to several ovarian cancer cell lines. Cell proliferation, as assessed using an ATP-based cell viability assay (CellTiter-Glo), was strongly inhibited in all ovarian cancer cell lines after HSP90 inhibition by 17-AAG (Figure 5A). Treatment with 17-AAG showed more profound anti-proliferative effects at day 6 than day 3. Cell proliferation IC50s at Day 6 were 350 nM for SKOV3, and 100 nM for OVCA429, and 750 nM for ES2, suggesting that 17-AAG anti-proliferative effects are more pronounced in ovarian cancer cells with multiply RTK activation (SKOV3 and OVCA429) than in ovarian cancer with single RTK activation (ES2). HSP90 inhibition also suppressed the expression of proliferation cell nuclear antigen (PCNA) proliferation marker in all three ovarian cancer lines; no apparent change of p53 expression was detected in these cells (Figure 4C).

The 24 or 48 hour 17-AAG treatments induced apoptosis, as evidenced by an increase of caspase3/7 activity (Figure 5B), the expression of caspase 8, and PARP cleavage (Figure 4C). Ovarian cancer lines analyzed at 48 h post 17-AAG treatment had dramatic increase in apoptotic cells compared to matched vehicle-treated cells (Figure 5C and Additional File 1 Table S1). The apoptosis was most prominent in SKOV3, the same cell line showing the highest level of nuclear fragmentation after 17-AAG treatment (Figure 5D).

Cell cycle analyses demonstrated a dose-dependent G2/G1 block with decreased S-phase population, and increased apoptotic portion (pre-G1 peak) in cells treated with HSP90 inhibitior 17-AAG (Figure 5D). Cell-cycle analysis in SKOV3 and OVCA429 showed a G2 block after HSP90 inhibition with an increase in the G2 peak from 12% and 10% in control cells to 24% and 20% after 17-AAG treatment, respectively (Figure 5D). This was accompanied by a decrease in the S-phase population from 14% and 13% of control cells to 8% of 17-AAG treated cells, respectively. ES2 cells showed a mild G1 block after HSP90 inhibition with an increase in the G1 peak from 74% of control to 78% of 17-AAG-treated cells (Figure 5D).

### HSP90 inhibition by a novel and pharmacologically favourable agent, AUY922, in ovarian cancer

AUY922 is a novel isoxazole-based HSP90 inhibitor, causes the degradation of multiple oncogenic cellular proteins and preclinical data suggest broad antitumor activity [32]. Because AUY922 has likely clinical advantages compared to 17-AAG, we evaluated AUY922 on RTK expression, RTK activation cell-cycle checkpoint protein expression, cell viability and apoptosis.

**Figure 6:**
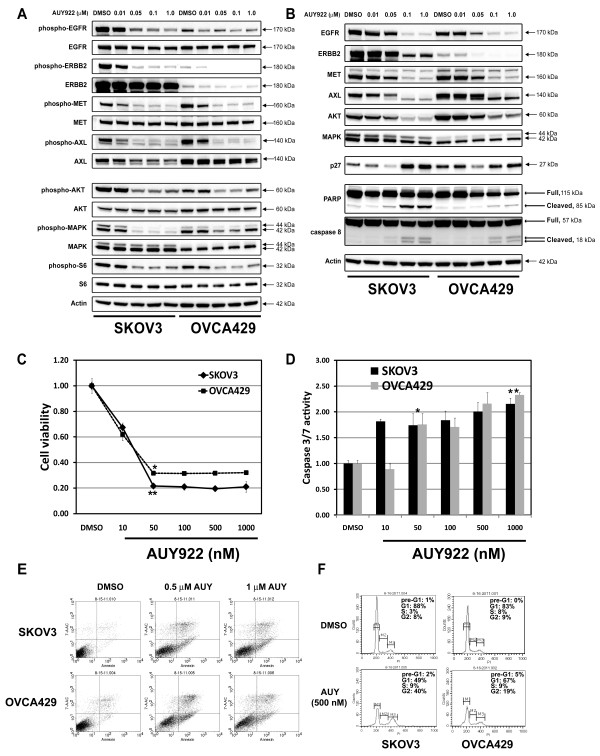
**Biochemical effects of HSP90 inhibitor AUY922 in ovarian cancer cell lines**. **A**) EGFR, ERBB2, MET, AXL, AKT, MAPK and S6 are inhibited, after 4 h AUY922 treatment in serum-free medium. β-actin stain is a loading control. **B**) Inhibition of EGFR, Erbb2, MET, AXL and AKT expression, with concomitant upregulation of p27, and variable Caspase 8 and PARP cleavage, after 48 h AUY922 treatment in serum-containing medium. β-actin stain is a loading control. **C**) Ovarian cancer cell viability was determined by the CellTiter-Glo assay after 72 h treatment with HSP90 inhibitor, AUY922. The data were normalized to the DMSO control, and represent the mean values (± s.d.) of quadruplicate cultures (*p < 0.05, **P < 0.01, n = 3). **D**) Apoptosis was evaluated in ovarian cancer cell lines, at 72 h after treatment with AUY922. Caspase 3/7 activity was measured using a Caspase-Glo luminescence assay. The data were normalized to the DMSO control, and represent the mean values (± s.d.) from quadruplicate cultures (*p < 0.05, **P < 0.01, n = 3). **E**) Apoptosis analyses following AUY922 (0.5 and 1 μM) treatment for 48 hours by using PE Annexin V Apoptosis Detection Kit I. **F**) Cell cycle analyses after 48 hours inhibitor treatment in serum-containing medium.

SKOV3 and OVCA429 were incubated with AUY922 for 48 h and subjected to western blot analyses. The phosphorylation of EGFR, ERBB2, MET, AXL, AKT, MAPK and S6 were all inhibited (Figure 6A); the total EGFR, ERBB2, MET, AXL and AKT expression were also inhibited (Figure 6B). These alterations were associated with upregulation of p27 (Figure 6B), consistent with cell cycle arrest induced by AUY922. Substantial reduce in cell viability was detected in both ovarian cancer cell lines by AUY922 (Figure 6C), and apoptosis was evidenced by caspase 8, and PARP cleavage, a significant increase in caspase 3/7 activity (Figure 6B and 6D), and a dramatic increase in apoptotic cells compared with matched vehicle-treated cells (Figure 6E and Additional File 1, Table S1). Cell cycle analyses demonstrated a G2 block in SKOV3 and OVCA429 treated with AUY922 (Figure 6F).

## Discussion

Ovarian cancer has the highest mortality rate of all gynecologic malignancy. Available therapies, including surgery, radiation, and chemotherapy, have not substantially improved survival for patients with ovarian cancer. Thus, there is an urgent need to further characterize ovarian cancer biologically and validate novel targeted therapies. Although the increasing evidence indicates tyrosine kinase activation promotes biological progression from nonneoplastic mesothelial lining of the ovaries or the fallopian tube epithelium to epithelial ovarian cancer, the clinical trials of small molecular tyrosine kinase inhibitors and monoclonal antibodies to RTK in patients with ovarian cancer failed to demonstrate clinical benefit [2]. For example, treatment of ovarian tumors with anti-EGFR or PDGFR agents had little response [33,34]. The reasons for this lack of efficacy of anti-RTK agents in ovarian cancer are unknown.

In our initial studies, we have evaluated the phosphorylation and expression of RTKs in individual ovarian cancer cell lines and primary frozen tumors (Figures 1 and 2). Our results suggested that the simultaneous activation of multi-RTK (EGFR, ERBB2, ERBB4, MET and/or AXL) in individual ovarian cancer contribute to the drug resistance to individual RTK inhibitors in ovarian cancer. Our results are in line with a recent study showing that cells with coactivation of RTKs were resistant to chemotherapy [35].

MET is highly expressed at different stages of neoplastic progression and capable of inducing the proliferation of ovarian cancer cells [36]. Several studies confirmed the important role of HGF/MET signaling in the transformation of surface ovarian epithelial cells and in the growth and dissemination of ovarian cancer [37,38]. Blocking the MET signaling by the MET inhibitors, PF-2341066, or by specific MET RNAi had antiproliferative effects and reduced tumor metastasis in ovarian cancer cells, possibly by suppressing MET-dependent PI3-K/AKT and RAF/MAPK signaling pathways [13,14]. In our present study, PHA-665752, a MET inhibitor, had mild effect in OVCA429 cell viability (Figure 3A), and PHA-665752 inhibition of viability did not correlate with baseline MET tyrosine phosphorylation in ovarian cancer (Figure 1 and 2B). Similarly, only a mild effect on ovarian cancer viability were detected after gefitinib-mediated EGFR inhibition and the cell death did not correlate with baseline EGFR tyrosine phosphorylation (Figure 1 and 2A, 2B and 2C), in spite of strong EGFR expression in many ovarian cancers (Figure 2A and 2C) [6,7]. Our findings are in consistent with the lack of efficacy of gefitinib or erlotinib in ovarian cancer clinical trials [3]. The combination inhibition of EGFR and MET by gefitinib and PHA665752 (1 μM) had similar anti-proliferative effects to the inhibition by each of RTKs (Figure 3A). AXL is another receptor tyrosine kinase known to be involved in ovarian cancers; AXL promoted proliferation in glioma cells and breast cancer cells [39,40], and AXL upregulation and activation was detected in ovarian cancers over normal ovaries (Figure 1 and 2) [22]. Our studies showed that AXL knockdown by RNA interference inhibited cell proliferation by 65% in OVCA429 cells, and the combination inhibition of EGFR, MET, and AXL inhibition resulted in 75% decrease in cell viability (Figure 3A).

HSP90 inhibition has shown anti-proliferative effects against ovarian preclinical models [41-43], however, the molecular mechanisms are unclear. Our studies show that multiple receptor tyrosine kinases are co-activated in individual ovarian cancer cells. The HSP90 inhibition led to the dephosphorylation and degradation of EGFR, ERBB2, ERBB4, MET and AXL in various ovarian cancer cells. Our studies showed that the phosphorylated forms of the RTKs were more sensitive to HSP90 inhibitor-mediated degradation (Figure 4A, B and 6A). Many protein kinases are degraded by a phosphorylation-dependent ubiquitin-proteasome system (UPS) [44]. CDC37, a co-chaperone of HSP90, stabilizes client proteins following their interaction with HSP90 and regulates protein kinase activity [45]. Treatment with HSP90 inhibitors such as 17-AAG or AUY922 led to UPS-dependent degradation of activated RTKs and total RTKs in a time-dependent manner, as those seen in GISTs and mesothelioma with HSP90 inhibition [46,30]. Moser C, et al. also pointed out the cancer selectivity and antitumoral effects of HSP90 inhibitors are regulated by affecting multiple targets and pathways, and identification of biomarkers such as RTK will be crucial for successful design and monitoring of targeting HSP90 therapies [47]. Furthermore, inhibition of HSP90 affects the tumor microenvironment by medicating non-malignant cells, such as endothelial cells and pericytes [47].

HSP90 inhibition by 17-AAG or AUY922 induced G1/G2 arrest and dramatic cell apoptosis (Figure 4C and 5B-D; Figure 6B and 6D-F). While treatment with 17-AAG induced the most markedly apoptosis in SKOV3 (Figure 5B, Figure 5C and Additional File 1, Table S1), AUY922 induced dramatic apoptosis in both SKOV3 and OVCA429 cells (Figure 6D, E and Additional File 1 Table S1).

The HSP90 inhibitor had a similar or greater anti-proliferation effect on various ovarian cancer cells compared to the combination inhibition of multiple RTKs (Figure 3A). Our studies also showed that individual RTK inhibitors have little or mild effect on ovarian cancer cell viability (Figure 3A). Taking together, these results suggested that the drugs targeting multiple RTK signaling simultaneously such as HSP90 inhibitors may be more effective in the treatment of ovarian cancer. So far, thirteen HSP90 inhibitors have been tested in clinical trial evaluation [48,49]. Although the HSP90-targeted drugs are currently not approved for clinical use, considerable progress has been made on various tumors trails including metastatic melanoma, multiple myeloma, non-small cell lung cancer, and leukaemia [48]. The HSP90 inhibitor 17-AAG has substantial activity against various human cancers in pre-clinical models by selectively degrading HSP90-client oncoproteins [50,51]. 17-AAG is now in Phase III validation with an improved formulation that overcomes several toxicities [49]. Several chemically different HSP90 inhibitors with improved oral biological availability have also been testing in clinic trial or will enter clinical trails [48]. Our current studies provided a mechanistic basis for the use of HSP90 inhibitors in ovarian cancer therapy.

Common downstream signaling of multiple RTK activation include the activation of PI3-K, mTOR and MEK, which play key roles in regulating survival, protein translation, and proliferation, respectively. In addition, these key signaling intermediates are also involved in differentiation, tissue invasion, angiogenesis, cell size, and cell responses to nutrients [52-54]. We have studied the activation of PI3-K, mTOR and MEK signaling in ovarian cancer cells treated with HSP90 inhibitor. HSP90 inhibition resulted in the inactivation of the AKT, S6, and MAPK (Figure 4B and 6B), which dramatically decreased cell viability by inducing cell apoptosis and G1/G2 cell cycle arrest (Figure 5, and 6) in each ovarian cancer cell line. Although p53 mutation plays the central roles in the molecular pathogenesis of high grade serous carcinoma [23], the expression of wild type (OVCA429) and mutant p53 (clear cell line ES2) was not affected after HSP90 inhibition by 17-AAG (Figure 4C).

## Conclusions

Our studies demonstrated that simultaneous activation of multi-RTKs including EGFR, ERBB2, MET, and AXL contributes to ovarian cancer cell proliferation and survival. HSP90 inhibition led to the inactivation of these receptor tyrosine kinases and suppress the downstream survival/proliferation signaling. These studies suggest that anti-multiple RTK strategy could be useful in the treatment of ovarian cancer.

## Supplementary Material

Additional File 1 **Apoptosis analyses (%) after 17-AAG and AUY treatment in ovarian cancer cell lines (SKOV3, OVCA429, and ES2)**. Apoptosis analyses following 17-AAG (0.5 and 1 μM) and AUY922 (0.5 and 1 μM) treatment for 48 hours by using PE Annexin V Apoptosis Detection Kit I. 17-AAG or AUY922 treatment had dramatic increase in apoptotic cells compared to matched vehicle-treated cells. Click here for file
