# Supplementary material for: Targeting HSP90 in ovarian cancers with multiple receptor tyrosine kinase coactivation
Source: Mol Cancer. 2011 Sep 30;10:125. doi: 10.1186/1476-4598-10-125 (PMC3196924; doi:10.1186/1476-4598-10-125)
Supplement: Additional File 1 — Apoptosis analyses (%) after 17-AAG and AUY treatment in ovarian cancer cell lines (SKOV3, OVCA429, and ES2). Apoptosis analyses following 17-AAG (0.5 and 1 μM) and AUY922 (0.5 and 1 μM) treatment for 48 hours by using PE Annexin V Apoptosis Detection Kit I. 17-AAG or AUY922 treatment had dramatic increase in apoptotic cells compared to matched vehicle-treated cells. [file 1476-4598-10-125-S1.DOC]

Table S1 Cell apoptosis analyses (%), as shown in Figure 5C and 6E, after 17-AAG and AUY treatment.

| **Inhibitor** | **SKOV3** | | | | **OVCA429** | | | | **ES2** | | | |
| --- | --- | --- | --- | --- | --- | --- | --- | --- | --- | --- | --- | --- |
|  | **UL** | **UR** | **LL** | **LR** | **UL** | **UR** | **LL** | **LR** | **UL** | **UR** | **LL** | **LR** |
| **DMSO** | 1.27 | 2.38 | 95.35 | 1.0 | 2.62 | 6.66 | 88.74 | 1.98 | 4.46 | 8 | 85.82 | 1.72 |
| **17-AAG (0.5 μM)** | 7.06 | 22.23 | 58.86 | 11.85 | 4.16 | 11.77 | 72.77 | 11.30 | 3.85 | 15.83 | 75.22 | 5.10 |
| **17-AAG (1 μM)** | 5.85 | 23.21 | 54.42 | 16.52 | 2.65 | 17.15 | 62.79 | 17.41 | 4.32 | 16.41 | 74.48 | 4.79 |
| **AUY922 (0.5 μM)** | 4.89 | 14.95 | 68.4 | 11.76 | 3.88 | 22.6 | 57.65 | 15.87 |  |  |  |  |
| **AUY922 (1 μM)** | 3.78 | 13.81 | 69.91 | 12.5 | 7.2 | 19.72 | 57.99 | 15.09 |  |  |  |  |
